# Precision percutaneous coronary intervention

**DOI:** 10.1038/s44325-026-00111-y

**Published:** 2026-03-23

**Authors:** Travis M. Wilson, Uzair Munaf, Nafhat Shaikh, Affan Rizwan, Riyan Siddiqui, Hafeez Ul Hassan Virk, Mahboob Alam, Umair Khalid, Muzamil Khawaja, Tanawat Attachaipanich, Larisa H. Cavallari, Tania Ahuja, Michael G. Nanna, Samin K. Sharma, Lloyd W. Klein, Gary S. Mintz, Chayakrit Krittanawong

**Affiliations:** 1 Department of Internal Medicine, Emory University School of Medicine, Atlanta, GA USA; 2 Department of Internal Medicine, University of Mississippi Medical Center, Atlanta, GA USA; 3 Department of Medicine, Liaquat University of Medical & Health sciences, Jamshoro, Pakistan; 4 Department of Internal Medicine, Baylor College of Medicine, Houston, TX USA; 5 Harrington Heart & Vascular Institute, Case Western Reserve University, University Hospitals, Cleveland Medical Center, Cleveland, OH USA; 6 The Texas Heart Institute, Baylor College of Medicine, Houston, TX USA; 7 Michael E. DeBakey VA Medical Center, Section of Cardiology, Baylor College of Medicine, Houston, TX USA; 8 Department of Cardiology, Emory University School of Medicine, Atlanta, GA USA; 9 Department of Internal Medicine, University of Missouri-Kansas City School of Medicine, Kansas City, MO USA; 10 Department of Pharmacotherapy and Translational Research and Center for Pharmacogenomics and Precision Medicine, University of Florida College of Pharmacy, Gainesville, FL USA; 11 Department of Pharmacy, NYU Langone Health, NYU Grossman School of Medicine, New York, NY USA; 12 Department of Medicine, Section of Cardiovascular Medicine, Yale School of Medicine, New Haven, CT USA; 13 Cardiac Catheterization Laboratory of the Cardiovascular Institute, Mount Sinai Hospital, New York, NY USA; 14 Department of Medicine, Section of Cardiology, University of California San Francisco, San Francisco, CA USA; 15 Cardiovascular Research Foundation, New York, NY USA; 16 HumanX, Delaware, DE USA

**Keywords:** Interventional cardiology, Acute coronary syndromes

## Abstract

Precision-based percutaneous coronary intervention (PCI) integrates contemporary strategies across the pre-, intra-, and post-procedural phases to improve outcomes and minimize complications. Emerging evidence underscores the value of these strategies in reducing adverse events and improving procedural efficiency. While artificial intelligence and pharmacogenomics hold long-term promise for enhancing personalization, their clinical utility in PCI remains in the early stages of development.

## Introduction

Contemporary treatment of cardiovascular disease (CVD) is rapidly evolving, with emerging research introducing the potential implementation of precision medicine. Precision medicine involves a focus on highly individualized patient care with novel risk stratification tools, individualized and targeted gene-specific and gene-editing diagnostics and therapies via pharmacogenomics, and more. Since CVD remains a leading cause of morbidity and mortality worldwide, evidence-based, personalized precision medicine supported by novel research is paramount, especially in the realm of coronary artery disease (CAD) and percutaneous coronary interventions (PCI). Precision PCI has become increasingly relevant in this realm. Precision PCI, which refers to a highly targeted and individualized approach to coronary revascularization, differs from traditional PCI in that it aims to leverage advanced imaging and physiological assessment tools to enhance the accuracy and effectiveness of PCI by utilizing technologies such as optical coherence tomography (OCT), intravascular ultrasound (IVUS), and fractional flow reserve (FFR) to guide decision-making and procedural execution. Further, artificial intelligence (AI)-guided PCI is a subset of precision PCI that leverages artificial intelligence or machine learning for lesion assessment, procedural planning, or real-time decision support, though its application remains largely investigational and is not yet broadly applicable nor available^1^. Within the field of Interventional Cardiology, specifically, precision care can incorporate contemporary data regarding intra-procedural and peri-procedural management for PCI to maximize patient comfort and satisfaction, optimize clinical outcomes, and minimize complications. The use of an individualized strategy for optimization of specific pre, intra, and post-PCI aspects of care may represent the future of a precision-based model of care as recent studies on such individualized care strategies reveal lower rates of post-PCI complications, mortality, and even shorter hospital stays, ultimately translating to higher patient satisfaction and lower healthcare system costs^2^. This contemporary review aims to summarize the available literature exploring the use of precision medicine when applied to pre, intra and post-procedural aspects of care for PCI patients. We conducted a targeted narrative review of the literature including English-language studies published between 1970 and 2025. Search terms included *percutaneous coronary intervention*, *precision medicine*, *intravascular imaging*, *fractional flow reserve*, *artificial intelligence*, and *peri-procedural management*. Priority was given to randomized controlled trials, systematic reviews, guideline statements, and high-quality observational studies, though smaller illustrative studies were included where evidence was limited. Reference lists of relevant articles were also screened to identify additional publications.

## Pre-procedural considerations

The pre-procedural period for PCI typically involves careful patient preparation to minimize risks of adverse events during and after the procedure. These considerations can be broadly categorized into procedural planning, risk stratification, and medication management.

## Procedural planning

Pre-procedural fasting has traditionally been recommended to reduce sedation-related risks such as aspiration and the potential need for intubation^3^. However, the risk of such an occurrence is extremely low, and the evidence supporting this practice in the modern era is limited^4^. Two randomized studies, the CHOW NOW (Can we Safely Have Our Patients Eat With Cardiac Catheterization - Nix Or Allow) and TONIC (Comparison Between Fasting and no Fasting Before Interventional Coronary Intervention on the Occurrence of Adverse Events) trials, have demonstrated the safety of a non-fasting strategy prior to cardiac catheterization with regards to nausea, vomiting, aspiration, and acute kidney injury (AKI), as well as 30-day mortality and readmission^5,6^. While patient satisfaction was similar between fasting and non-fasting groups in these trials, this evidence suggests room for personalized approaches based on patient preference and procedural risk. It is important to note, however, that these findings may not generalize to higher-risk coronary interventions, including those involving mechanical circulatory support or atherectomy, where the risk of hemodynamic instability and potential need for deeper sedation or general anesthesia remains significant. Therefore, fasting protocols should be carefully individualized in such cases. The ongoing SCOFF (Safety and Care OF no Fasting) trial will further assess the efficacy and impact of a non-fasted strategy^7^.

## Risk stratification

AI is rapidly transforming pre-PCI preparation and guidance, particularly in the realm of CCTA diagnostics and pre-procedural assessment. For example, AI-based machine learning has been employed in tandem with fractional flow reserve computed tomography for segmenting coronary geometry and plaque prior to PCI. This has been demonstrated to improve diagnostic performance of the identification of hemodynamically significant lesions in a study conducted by Nørgaardet et al.^8^. This offers a non-invasive assessment of coronary physiology which provides a comprehensive physiological map, enabling the identification of functional CAD patterns, informing revascularization strategies. A number of groups have introduced AI systems that have proven useful in detection of CAD stenosis pre-procedurally as well. Zreik et al. developed two models that aimed to distinguish patients with and without obstructive CAD which proved to be 85% and 95% accurate per patient and per vessel assessed, respectively^9^. Further, Kang et al. has introduced a two-step AI algorithm which has proven to be 95% specific and 93% sensitive in detecting CAD stenosis within one second of assessment^10^. Various other studies have proposed models that have shown similar efficacy in evaluation of Coronary Calcium Scoring as well as plaque characterization using AI as well, all of which offer exciting potential to significantly inform and improve PCI planning in the years to come^11,12^.

## Medication management

Managing peri-procedural anticoagulation is another critical component of pre-procedural preparation, typically tailored to the nature of the planned procedure. Up to 10% of patients referred for PCI are taking an oral anticoagulant (OAC)^13^. For routine coronary angiography, OAC is often held for 24–48 h (or longer if vitamin K antagonists [VKA] are used), particularly when femoral access is planned^3^. Interruption of OAC may increase the risk of thromboembolic complications while the use of bridging may inadvertently increase peri-procedural bleeding risk^14,15^. In the largest real-world analysis from the SWEDEHEART (Swedish Websystem for Enhancement of Evidence-Based Care in Heart Disease Evaluated According to Recommended Therapies) registry, researchers assessed the effects of interruption versus no interruption of OAC in patients admitted for PCI or an intracoronary diagnostic procedure. The study included patients receiving either VKA or OAC therapy with nearly 35% undergoing femoral access. Results of the study demonstrated that the uninterrupted OAC group had no significant differences in rates of major adverse cardiac and cerebrovascular events (MACCE) at 120 days, nor in major or minor bleeding. This group also was shown to have a shorter median duration of hospital stay^13^. While bleeding and thrombotic risk should be assessed at an individual level with consideration of both the patient and the characteristics of the procedure, a strategy of uninterrupted OAC may be considered, as supported by large-scale real-world data. Nevertheless, additional studies and data on uninterrupted anticoagulation prior to PCI are warranted.

## Intra-procedural factors

### Sedation

Procedural sedation strategies during PCI play an important role in ensuring safety, enhancing patient comfort, managing vascular access-related events such as vasospasm, and possibly impacting pharmacodynamics peri-procedurally. In the context of precision PCI, sedation is not merely a matter of routine practice but rather an opportunity for individualized optimization. Tailoring sedation to patient-specific factors, such as anxiety tolerance, comorbidities, and risk for vasospasm or oversedation, aligns with the broader goals of precision PCI by improving procedural efficiency, minimizing complications, and enhancing the overall patient experience.

Routine and urgent cardiac catheterization are typically performed with conscious sedation and local anesthetic in North America^16^. However, pre-medication may be considered. One study demonstrated that a single pre-procedural dose of diazepam reduced reported peri-procedural pain and was found to have no adverse effect on vascular access-site safety outcomes^17^. Local anesthetic-only strategies have also been considered. However, the optimal choice for sedation seems to include a combination therapy of topical lidocaine and intravenous midazolam which has been shown to reduce radial artery spasm, arterial puncture attempts and time, access-site crossover, as well as patient discomfort, when compared to neither or either medication alone^18^. The combination of opioids and benzodiazepines, particularly fentanyl and midazolam, has also been shown to reduce arterial vasospasm, access site crossover, and patient discomfort when compared to no treatment^19^. Varying the timing between initial sedation and administration of local anesthetic has notably been found to have no differences in total pain medication use, patient satisfaction, or pain assessed by either patient or nurse during cardiac catheterization^20^.

In the setting of acute coronary syndrome (ACS), there are important factors to consider in regard to sedation choice. For instance, morphine is thought to delay the onset of action for ticagrelor as demonstrated in the ATLANTIC (Administration of Ticagrelor in the Cath Lab or in the Ambulance for New ST Elevation Myocardial Infarction to Open the Coronary Artery) trial^21^. Similarly, the PACIFY (Platelet Aggregation With Ticagrelor Inhibition and Fentanyl) trial, which randomized patients undergoing elective PCI to receive fentanyl or no treatment prior to loading with ticagrelor, found that fentanyl delayed ticagrelor’s antiplatelet effect^22^. The opioid-P2Y12 inhibitor interaction is thought to result from delayed gastric absorption of the drug. To combat this issue, utilization of gastric motility agents such as metoclopramide may be considered^23^. Alternatively, strategies to minimize or avoid sedation can be employed. For example, there is evidence that patient-selected music may reduce the need for pharmacologic sedation during cardiac catheterization^24^.

The data available on sedation strategies during cardiac catheterization, while limited, offer insight into the potential for individualizing sedation on a patient-to-patient basis. This would require consideration of several important patient factors such as patient pain and anxiety tolerance, risks for vasospasm (females, smaller vasculature), risks for oversedation (critically ill, elderly, high risk for aspiration, renal or hepatic disease) as well as patient preference. Additionally, the minimization and avoidance of pharmacologic interactions of sedatives with antiplatelet agents are tantamount in the setting of drug-eluting stent implantation PCI for prevention of stent-related thrombosis. There are a number of studies that suggest that pharmacogenomics may aid in guiding sedation selection for individual patients. For instance, one study conducted by Yağar et al. demonstrated that the ADRA2A C1291G polymorphism affects sedative and hemodynamic responses in patients undergoing coronary artery surgery^25^. Further, a study conducted by Ding et al. identified nine single nucleotide polymorphisms involved in transport proteins, metabolic enzymes, and target proteins specifically in Dexmedetomidine that are likely related to variability in sedative and hemodynamic effects of the medication^26^. Additional studies are warranted to further validate the clinical utility of pharmacogenomic assessments in individualized selection of sedation for patients in the future.

### Vascular access and complications

Optimizing sedation strategies and procedural decisions, such as the site of vascular access, are crucial for patient outcomes and procedural efficiency in PCI. Access site selection typically depends on a factors such as patient anatomy procedure type, bleeding risk, anticoagulation status, and operator experience. A transradial access (TRA)-first approach is recommended and supported by randomized studies and meta-analyses demonstrating reduced bleeding risk, improved vascular outcomes, lower mortality, and greater patient comfort when compared to transfemoral access (TFA)^27,28^. Alternative vascular access sites, such as brachial, distal radial, and ulnar artery access, are possible, though infrequently utilized for PCI. Brachial arteriotomy is a well-established approach that has been well-described in decades prior, though it is associated with considerable vascular complication rates that often require surgical repair^29,30^. In cases of anatomical limitations, such as radial artery harvesting for bypass grafting or peripheral arterial disease, transbrachial access (TBA) may be employed and has shown similar rates of vascular complications to TFA with the added benefit of a nearly 55% bleeding risk reduction in contemporary meta-analyses^31^. However, compared to TRA, TBA results in higher rates of major entry site complications^32^. Distal radial access, which was introduced in 2017, involves accessing the radial artery via the anatomical snuffbox, and is associated with lower rates of radial artery occlusion at the expense of nearly twice the rate of access failure^33^. Ulnar access is slightly inferior to TRA with regards to cannulation time, procedural success rate, and crossover, though it is significantly more effective when compared to distal radial access^34^. Prior to determining access, it is important to consider that TRA precludes future utilization for bypass grafting which may be a key decision-making point in certain patient populations. Ultimately, transradial access appears to be the most optimal choice for upper extremity access, though distal radial access, once mastered, may reduce rates of vascular compromise^29–34^.

In patients requiring eventual coronary revascularization via internal mammary artery (IMA) grafting, use of the ipsilateral radial artery is recommended to facilitate IMA angiography; however, left radial access may require multiple catheter exchanges for complete native and graft angiography and increasess the risk of vasospasm^35^. Right radial access for left IMA (LIMA) angiography is technically possible with multiple catheters, but it involves navigating a catheter through the aortic arch, cannulating and advancing the wire and catheter through the left subclavian artery, and ultimately selectively engaging the IMA. Left subclavian cannulation may also be achieved via a right TRA with several catheters^35,36^. A blood pressure cuff can even be utilized to externally compress a wire to facilitate catheter advancement and exchange, or to assist in nonselective angiography of the IMA in cases of difficult engagement. Overall, varying success rates are described for LIMA angiography via right TRA, ranging from approximately 60% to nearly 90%^37,38^. Based on meta-analyses of randomized data, routine use of right versus left TRA is associated with slightly longer fluoroscopy time and increased contrast use^39,40^. Increased tortuosity and potentially more difficult catheter manipulation due to innominate-aorta angulation in the right subclavian artery, compared to the left, may account for these findings. Interestingly, in graft angiography and intervention, TRA, when compared to TFA, showed increased contrast volume, longer procedure time, greater patient and operator radiation exposure, and approximately 20% more access crossover albeit with similar rates of vascular complications^41^.

As part of optimizing vascular access, ultrasound guidance is recommended in all catheterization labs^27^. Utilizing ultrasound guidance for TRA improves first-pass success and reduces the risk of access failure^42^. Additionally, ultrasound guidance also reduces vascular access site complications and major bleeding in TFA and improves chances of success with vascular closure devices^43^. Studies have shown that operators may develop a level of proficiency and comfort with ultrasound that significantly reduces complications rates after as few as twenty ultrasound-guided cases^44^.

More novel approaches in the realm of precision imaging and simulation tools have begun to be developed to improve PCI access decisions. Notably, three-dimensional (3D) virtual reality (VR) guiding catheter simulations have been noted in two select cases by Yoshinaga et al. They detail the use of a VR simulation system based on 3D rendering reconstructions of CT imaging data as a tool for pre-procedural planning that effectively informed the selection of appropriate guiding catheters and vascular access without patient exposure to radiation or radiocontrast^45^. Alternative approaches have been proposed by Yao et al. in which, they have developed a novel PCI guidance system by combining a vascular segmentation network and heuristic intervention path planning algorithm based on a dataset of digital subtraction angiography images. They report that this system offered 95.8% accuracy for reasonable and satisfactory paths for PCI^46^. Virtual coronary intervention has also been proposed as a means of predicting a patient’s physiological response to stenting, not only allowing for pre-procedural approach but accurately estimating virtual FFR (vFFR) as an approximation of post-procedural measured FFR^47^.

Though ultrasound and other innovations have improved vascular access, TRA comes with its own set of challenges. One pitfall of TRA includes the increased rate of vasospasm, which can increase procedural difficulty, require access-site crossover, or even preclude procedural success. Typically, an intra-arterial radial “cocktail” of vasodilatory medications, including calcium channel blocker and/or nitrate, is administered to reduce this risk. However, one meta-analysis showed that neither topical nor intra-arterial nitroglycerin is effective in preventing vasospasm or radial artery occlusion alone, while subcutaneous administration was demonstrated to reduce risk of both complications^48^. In another small trial, verapamil added to intra-arterial heparin and nitroglycerin was also shown to be effective in reducing radial artery occlusion^49^. In contrast, for distal radial access, addition of verapamil to nitroglycerin alone did not significantly impact spasm or patient discomfort during angiography^50^.

In regions where these typical agents may be unavailable, a trial comparing intra-arterial nicardipine and isosorbide dinitrate (ISDN) versus ISDN alone demonstrated vasospasm reduction and reduced femoral crossover, offering another viable strategy^51^. Antithrombotic therapy, usually with heparin, can also be utilized to reduce radial artery occlusion. Even bivalirudin has been tested as an alternative antithrombotic to heparin by Plante et al. but it does not appear to significantly alter rates of radial artery occlusion^52^. However, this study was limited by its non-randomized design.

While the site of vascular access remains one of the key decision points in procedural efficacy and complication reduction, vascular sheath selection also plays a role. Larger sheath diameters, in particular the use of a 6 French in comparison to a 5 French sheath, increase radial artery occlusion rates, though rates of hemorrhage, pseudoaneurysms, and arteriovenous fistula formation are similar^53^. Sheath length does not appear to have the same impact on outcomes, though hydrophilic sheaths have been shown to reduce spasm and discomfort^54^. Similarly, thin-walled (Slender) sheaths have been shown to not impact access complication rates^55^. For patients with smaller vascular anatomy, a hydrophilic, sheathless guide catheter with a smaller diameter than typical introducer sheaths has been utilized safely and effectively for complex TRA PCI, as well as for distal radial and ST-elevation myocardial infarction (STEMI) interventions, without vascular access site complications^56,57^.

### Cardiac catheterization and percutaneous coronary intervention

A central tenet of precision PCI is the ability to integrate patient-specific hemodynamic, anatomic, and procedural factors into real-time decision-making. Several domains, such as left ventricular filling pressures, the technical challenges of PCI after transcatheter aortic valve implantation (TAVI), and the selection of mechanical circulatory support illustrate how individualized assessment directly influences procedural safety and outcomes.

#### Left ventricular end-diastolic pressure

One important component of PCI assessment is the measurement of left ventricular pressure, which has demonstrated significant predictive value in angiography. However, this practice is often overlooked. Even in elective PCI, an elevated LV end-diastolic pressure (LVEDP) above 18 mmHg (especially equal to or greater than 26 mmHg) is associated with increased risk of in-hospital death, acute heart failure, or AKI^58^. This suggests that LVEDP should be routinely measured and considered before proceeding with elective PCI. In a separate analysis, increased LVEDP (≥20 mmHg) was an independent predictor of contrast-induced AKI^59^. Given these findings, strategies such as LVEDP-guided volume expansion have been explored to mitigate the risk of contrast-induced AKI following cardiac catheterization^60^. Interestingly, some studies have found that the addition of low dose furosemide to hydration, particularly when guided by LVEDP, may also mitigate contrast induced-AKI risk^61,62^. By tailoring periprocedural hydration strategies to a patient’s LVEDP, such as volume expansion or LVEDP-guided use of low-dose diuretics, clinicians can personalize care to reduce contrast-related complications. These findings support the routine use of left heart catheterization, when possible, to provide useful hemodynamic data that can guide decision-making in cardiac catheterization and PCI, ultimately reducing adverse outcomes.

#### Transcatheter aortic valve implantation

Another patient factor that can alter a technical approach to coronary angiography and PCI is the presence of prior TAVI. A prior TAVI can make coronary angiography and PCI technically difficult, particularly with supracoronary devices such as the Medtronic Evolut and CoreValve, Abbott Portico, and Boston Scientific ACURATE valves^63^. These devices, when compared to subcoronary devices, such as the Edwards SAPIEN, JenaValve, and the discontinued Boston Scientific Lotus Edge, can make utilization of standard diagnostic catheters more difficult due to their height and positioning^64^. Supra-coronary devices may make coronary artery engagement more challenging due to obstruction of the catheter by the stent struts or the crown of the stent frame. Here, precision PCI involves anticipating anatomic and device-specific challenges, selecting appropriate catheters, and using adjunctive imaging tools such as CCTA to plan access strategies. For PCI, guide catheters should be used cautiously to prevent entrapment within the valve frame. It is recommended to cannulate the coronary ostia through the middle of the valve frame cell or from a cell above the level of the ostia^65^. The preferred guide catheters for left main engagement include IM and JL4, while an AR1 and JR 3.5 are often selected for engagement of the right coronary artery (RCA)^65,66^. When necessary, guide extension catheters and/or microcatheters can aid in successful engagement of the coronary ostia.

Previously, the technical difficulty of post-TAVI coronary access was less concerning due to the limited population requiring the intervention. Now that TAVI is offered to a broader demographic, greater care should be given in prosthetic selection and placement – particularly with regards to implantation height, depth, and sizing of the sinus of Valsalva. This raises the question as to whether PCI for concomitant CAD should be performed prior to or concurrently with TAVI. Currently, there is no consensus on optimal strategy in this scenario and the decision is often individualized based on a patient’s individual clinical profile^67^.

Notably, post-TAVI ACS has been associated with significantly higher rates of cardiogenic shock, cardiac arrest, and in-hospital mortality^68^. Pre-TAVI PCI may help reduce the peri-procedural ischemic risk during TAVI, related to rapid pacing and/or balloon inflation during valve deployment. However, randomized trials and meta-analyses comparing PCI with and without TAVI demonstrated no significant differences in clinical outcomes^69,70^. Coronary CT angiography (CCTA) has been offered as a tool that may help predict difficult coronary engagement to mitigate these risks^71^. Nonetheless, angiography and PCI following TAVI remains technically feasible, especially with the development of advanced imaging modalities.

One major factor to consider when pursuing PCI in TAVI patients is the decision-point between using side hole vs. non-side hole guide catheters. Guide catheters are typically selected based on the degree of support they provide during PCI. For engagement of the ostial left main and the RCA, catheters with side holes are often employed as they allow some degree of blood flow and minimize pressure dampening^72^. While this benefit allows for continuous blood flow when the catheter is deeply seated in the coronary ostium, there is some evidence that side hole guide catheters may compromise the level of support offered. This was noted in a prospective study of twenty-five patients who underwent clinically indicated FFR measurement for coronary artery stenosis. The results demonstrated a reduction in measured FFR among side hole catheters in comparison to non-side hole catheters^73^. Finally, there is some evidence that suggests the purported benefit of maintaining coronary perfusion via side holes may be a false reassurance, as the non-dampened pressures measured by side hole catheters have been shown to represent a measurement of aortic pressure rather than true coronary perfusion pressure^74^. Further research is warranted to elucidate whether side hole catheters confer a definitive benefit in clinical practice.

#### Circulatory support and percutaneous coronary intervention

For patients undergoing high-risk PCI, individualized hemodynamic support is another key domain of precision care. Recent data comparing intra-aortic balloon pump (IABP) support to microaxial flow pumps (such as the Impella) reveal a shift in preferred approaches for cardiac support for high-risk PCI. Prior data on IABP support for high-risk PCI were mixed, particularly when compared to the Impella^75^. The PROTECT II (Prospective Randomized Clinical Trial of Hemodynamic Support with Impella 2.5 vs. IABP in High-Risk PCI) trial demonstrated improved 90-day outcomes with the Impella 2.5 compared to IABP for patients with complex multivessel disease or unprotected left main disease (UPLMD) and severely reduced LV function^76^. More recent registry data also shows improved outcomes, including in-hospital survival, with Impella use compared to IABP use for nonemergent high-risk PCI^77^. An interim analysis of PROTECT III, comparing outcomes with PROTECT II, showed further improvement in MACCE outcomes at 90 days^78^. Overall, particularly in light of the benefits of Impella-supported PCI, the role of IABP-supported PCI has become increasingly limited.

Alternative advanced circulatory support mechanisms include the TandemHeart left ventricular assist device and venoarterial extracorporeal membrane oxygenation support (VA-ECMO). A number of observational studies have demonstrated that the TandemHeart is independently safe and effective in offering hemodynamic support in high-risk PCI^79,80^. However, a metanalysis comparing the efficacy of TandemHeart to that of the Impella demonstrated higher 30-day mortality rates among patients supported with the TandemHeart (8% versus 3.5%) as well as higher bleeding and vascular complication rates^81^. Currently, no randomized clinical trials exist to support the use of the TandemHeart in high-risk PCI.

VA-ECMO is a circulatory support modality that is a viable option for patients with pre-existing biventricular failure. The benefits of VA-ECMO lie in its ability to offer 100% of a patient’s cardiac output. There is little data to support the theoretical benefits of VA-ECMO as a circulatory support approach. Van den Buijs et al. conducted a retrospective study in which they compared hemodynamic support among complex high-risk PCI with VA-ECMO versus Impella which demonstrated no significant difference in periprocedural hemodynamic instability, MACE, bleeding complications, in-hospital mortality or 30-day mortality^82^. Though this study was significantly limited in power, noting to only have enrolled 14 patients who underwent PCI with VA-ECMO assistance. Further, there are a number of limitations that make VA-ECMO a less than ideal mode of support in this setting, including its inability to reduce ventricular wall stress without venting assistance^83,84^.

Taken together, LVEDP measurement, PCI after TAVI, and mechanical circulatory support highlight the breadth of intra-procedural considerations requiring precision. By integrating hemodynamic assessment, anatomic constraints, and individualized support strategies, interventionalists can tailor PCI to each patient’s unique profile, enhancing safety, optimizing outcomes, and advancing the goals of precision PCI.

#### Integrated Artificial Intelligence (AI)

AI, encompassing advanced computational technologies such as machine learning (ML) and deep learning (DL), is fundamentally transforming interventional cardiology, particularly in the PCI catheterization laboratory. These technologies enable precision medicine by tailoring care to each patient’s unique characteristics, leveraging large datasets and sophisticated algorithms to enhance decision-making, streamline workflow, and standardize procedural assessment.

A primary application of AI is the accurate and objective quantification of coronary lesion severity, moving beyond traditional 2D quantitative coronary angiography to 3D-QCA–based functional assessment^85^. Angiography-derived FFR and instantaneous wave-free ratio (iFR) use computational fluid dynamics or mathematical algorithms to estimate physiologic significance from coronary angiograms. Commercial examples include AutocathFFR (MedHub Ltd.), FFRangio (CathWorks), Quantitative Flow Ratio, vFFR, and Coronary Angiography-derived FFR (FlashAngio), all of which have demonstrated high sensitivity and specificity compared to invasive FFR^86,87^. Systems like Angio iFR (Philips Healthcare) also estimate non-hyperemic indices, identifying the physiologically most significant lesion in cases of diffuse or tandem disease^85^.

Beyond angiography, ML supports interpretation of physiologic data. For example, the CEREBRIA-1 study demonstrated that a computational ML program interpreting pressure-wire pullback data was noninferior to expert consensus in determining optimal PCI strategy^88^. Similarly, AI-driven software such as CathAI automates angiogram interpretation and stenosis severity estimation, improving standardization and reducing inter-operator variability^89^. These tools expedite analysis and promote the routine application of coronary physiology in clinical practice.

AI also improves planning and execution by processing high-resolution intravascular imaging modalities, such as OCT and IVUS, which historically required expert interpretation. Deep learning algorithms automate segmentation, quantify calcium burden (e.g., Ultreon 1.0), and characterize plaque composition (e.g., OctPlus), assisting in vessel preparation, stent sizing, and identification of high-risk lesions^90,91^.

Furthermore, AI-enabled 3D lumen reconstruction combined with computational flow dynamics allow derivation of intravascular imaging-based FFR metrics, such as Optical Flow Ratio and Ultrasonic Flow Ratio, providing combined anatomical and physiologic assessment^92^. Automated co-registration software, including SyncVision and iFR Scout, integrates intravascular or physiologic data with angiography to guide lesion localization, define stent landing zones, and optimize treatment strategy. Prospective data indicate that co-registration alters PCI strategy in over 40% of cases^93^. Nevertheless, an important unresolved issue arises when physiologic indices and plaque morphology are discordant. Lesions may appear physiologically insignificant but harbor high-risk features (thin-cap fibroatheroma, large lipid pools, spotty calcification), or conversely demonstrate physiologic significance without overt vulnerability. Current integration strategies remain variable, underscoring the need for further evidence to guide management in these scenarios.

Enhanced, AI-enabled visualization tools also support procedural execution. Dynamic Coronary Roadmapping overlays coronary anatomy on fluoroscopic images, aiding wire navigation and stent deployment while reducing contrast volume and risk of contrast-induced nephropathy^94^. Enhanced stent visualization systems augment stent visibility during fluoroscopy, supporting precise post-dilatation and minimize complications from overlapping stents^95,96^.

Finally, AI is accelerating the development of robotic-assisted PCI, enhancing procedural precision and operator safety. Systems such as CorPath GRX allow operators to perform procedures remotely, substantially reducing radiation exposure, and include automated movements like “Rotate on Retract” to optimize device navigation^97^. Reinforcement learning and learning from expert demonstration are being investigated to enable conditional task autonomy for catheter and guidewire manipulation, with proof-of-concept studies exploring autonomous or semi-autonomous PCI execution, and Tele-robotic interventions have been tested in pilot studies, offering potential for remote expert guidance and broader access to complex PCI^97^.

Collectively, AI in the PCI lab is driving precision care by enabling physiologically guided diagnosis, automated high-resolution intravascular imaging interpretation, optimized procedural planning, and enhanced procedural execution. These technologies reduce variability, aim to improve outcomes, and provide a foundation for future developments in robotics and autonomous intervention, marking a paradigm shift in interventional cardiology.

### Intravascular Imaging

Intravascular imaging is increasingly utilized to assess coronary lesions and to guide percutaneous interventions. This includes IVUS and OCT. The 2021 ACC/AHA/SCAI guidelines for coronary artery revascularization provide a Class IIa recommendation for the use of intravascular imaging to guide elective PCI and a Class Ia recommendation for imaging in the setting of ACS, similar to the 2024 European Society of Cardiology (ESC) Class Ia recommendation for intravascular imaging for anatomically complex lesions. These guidelines support intravascular imaging for pre-intervention assessment of plaque burden, extent of calcification, lesion length, and external elastic lamina diameter for stent sizing, as well as post-intervention evaluation of minimum stent area, malapposition, underexpansion, tissue protrusion, edge disease, and edge dissection^98,99^. Compared with angiography-guided PCI, intravascular imaging guidance for complex disease such as bifurcation lesions, UPLMD, severe calcific disease, ostial disease, multivessel disease, in-stent restenosis (ISR), chronic total occlusion (CTO), and extremely long lesions as a composite see improved outcomes, including survival^100^. Insights about plaque morphology can inform plaque modification strategies, such as the utilization of intravascular lithotripsy (IVL), directional or laser atherectomy, the use of noncompliant scoring or cutting balloons, or even proceeding with direct stent placement. The mechanism of ISR can also be further characterized with intravascular imaging, whether it be due to fibrotic neointimal hyperplasia, calcific ISR, neoatherosclerosis with lipid plaque ISR, or stent underexpansion – all of which may be managed by different, targeted interventions. The use of OCT and IVUS alters PCI strategy in the majority of patients in which it is employed, including in ACS cases, and guides post-PCI stent optimization^101^. Furthermore, the degree of lesion calcification impacts the risk of multiple aspects of procedural failure, but particularly stent underexpansion. IVUS- and OCT-derived lesion characteristics (circumferential calcium extent, calcium length, vessel size, calcific nodule, calcium thickness) are predictive of stent underexpansion and, when utilized effectively, allow for greater stent expansion with calcium modification^102^.

In addition to their role in the management of coronary lesions, IVUS and OCT play a pivotal role in the diagnosis and management of suspected spontaneous coronary artery dissection (SCAD). IVUS is exceptionally proficient in the identification of true and false lumens when confirming the diagnosis of SCAD, even when angiographic evidence may be inconclusive^103^. Additionally, IVUS offers the ability to differentiate intramural hematomas when angiographic appearance may present similarly to atherosclerosis or coronary spasm^104^. In contrast, OCT offers higher resolution for characterization of vessel walls, which proves useful in visualizing intimal tears, flaps, and intraluminal thombi^103–105^. When applied in unison, IVUS and OCT offer clinicians a comprehensive understanding of SCAD pathophysiology. Where IVUS provides a broader field of view and better assessment of the overall extent of dissection, OCT’s higher resolution allows for precise identification of the dissection entry point, aiding clinicians in making informed decisions regarding conservative versus aggressive management of SCAD.

Another condition for which intracoronary imaging is essential is myocardial infarction with non-obstructive coronary artery disease (MINOCA). OCT is particularly useful in this realm. Several studies have shown that OCT identifies the underlying etiology of MINOCA in a substantial proportion of cases where angiography demonstrated non-obstructive coronaries. One such retrospective study conducted by Yamamoto et al. found that 25% of patients who met these criteria were found to have abnormal findings on OCT (namely thrombus, plaque rupture, or intimal lacerations)^106,107^. When combined with cardiac magnetic resonance (CMR) imaging, OCT has significantly augmented diagnostic power. This was well-supported in a study by Reynolds et al. where integrated OCT and CMR successfully identified an etiology in 84.5% of patients with MINOCA^108^. While IVUS offers greater tissue penetration, and aids in evaluating plaque burden, it may not be as sensitive at OCT at detecting various MINOCA-related pathologies and is less often used in diagnosis^109^.

Overall, intravascular imaging should be routinely employed to optimize PCI and to mitigate the risk of short- and long-term procedural failure, such as PCI-related complications requiring further intervention, myocardial infarction (MI), or the later need for target lesion revascularization.

### Calcium modification techniques

Atherectomy for lesion modification comes in the form of excimer laser coronary atherectomy (ELCA), orbital atherectomy (OA), rotational atherectomy (RA), and most recently IVL. Head-to-head randomized data for these various techniques are lacking; however, each may be employed in a variety of situations to optimize lesions for stent deployment. ELCA is a less frequently utilized, older technology that ablates tissue by breaking molecular bonds, increasing plaque compliance, and cavitating soft plaque and thrombus which can be beneficial in fibrotic ISR or refractory thrombus, though less ideal for calcium modification^102^. It is often deployed prior to other forms of atherectomy if there is difficulty with delivering alternate devices across a lesion. RA uses a rapidly rotating diamond burr to debulk and fracture inelastic, calcific plaque to facilitate stent delivery and expansion and improves procedural success rates^110^. In contrast, OA utilizes an eccentrically placed diamond-coated crown as a means of debulking and fracturing plaque, functioning bidirectionally as a result of the eccentricity of its crown^111^. In a small OCT-based study, OA was demonstrated to create deeper dissections within plaque when compared to RA, leading to lower rates of stent malapposition^112^. Selection between RA and OA is often based on lesion location, vessel diameter, calcium eccentricity, and tortuosity of the target vessel. Often, RA is preferred in aorto-ostial lesions concentric calcific stenoses, smaller vessels, and in tortuous vessels as it can be delivered over a floppy wire, while OA is limited by its stiff wire^113^. Alternatively, IVL utilizes a balloon catheter that, when inflated and activated, creates pulses of vapor bubbles within the balloon that result in acoustic shockwaves with peak acoustic pressures of approximately 50 atmospheres^111^. The balloon itself is deployed at 4 atmospheres to allow for the lithotripsy energy delivery, but has a nominal pressure of 6 atmospheres and rated burst pressure of 10 atmospheres. This balloon may be deployed at higher pressures following IVL delivery to facilitate lesion expansion, though this strategy lacks high-quality evidence to guide practice.

### Pharmacology for PCI

Precision medicine in pharmacology during PCI is crucial for optimizing patient outcomes by tailoring antiplatelet and anticoagulant therapies to individual genetic, metabolic, and clinical profiles, thereby reducing the risks of adverse events and improving procedural success. Unfractionated heparin (UFH) and bivalirudin are two key intravenous anticoagulants for PCI. UFH has long been a cornerstone in catheterizations labs; however, challenges like variable anticoagulation, platelet activation, and heparin-induced thrombocytopenia limit its application^114^. Low molecular weight heparin (LMWH), particularly enoxaparin, offers more predictable anticoagulation without the need for continuous monitoring. Studies by Ali-Hasan-Sayegh et al. and the ATOLL (Acute Myocardial Infarction Treated with Primary Angioplasty and Intravenous Enoxaparin or UFH to Lower Ischemic and Bleeding Events at Short- and Long-term Follow-up) trial demonstrated intravenous enoxaparin’s superiority over UFH in achieving optimal thrombolysis in myocardial infarction (TIMI) flow and reducing repeat MI, with comparable bleeding risks^115,116^. The LOW-RAO (Low-Molecular-Weight Heparin in Radial Artery Occlusion Treatment) study evaluated LMWH’s efficacy in treating radial artery occlusion (RAO), revealing that 41.4% of patients in the LMWH group achieved improved radial artery patency compared to 10.3% in the control group receiving no treatment. Bleeding events were not significantly higher in the LMWH group, suggesting that weight-adjusted LMWH administered subcutaneously for up to 4 weeks improves radial artery patency without increasing bleeding complications. These findings support the clinical application of LMWH in PCI for enhanced efficacy and safety^117^.

Though LMWH’s efficacy is well supported, its safety profile, particularly regarding bleeding risk, has been debated as it is renally cleared and theoretically poses a potential for unpredictable anticoagulation in patients with impaired renal function. Several studies have explored this notion. Sabatine et al. found no significant difference in bleeding rates between LMWH and UFH, demonstrating a comparable safety profile across various subgroups, including those on clopidogrel and among those with reduced renal function^118^. Similarly, Lee et al. reported similar hemorrhagic rates between LMWH and UFH when used in conservative ACS management, with LMWH reducing risk of mortality and ischemia. However, in early invasive strategies, the adjunctive use of LMWH with clopidogrel and glycoprotein IIb/IIIa inhibitors (GPIs) was shown to increase major bleeding risk^119^. The SYNERGY (Superior Yield of the New Strategy of Enoxaparin, Revascularization and Glycoprotein IIb/IIIa Inhibitors) trial demonstrated a modest increase in bleeding with enoxaparin, but noted that it is likely superior to UFH when started as an initial first-line agent, given that it was not switched to an alternate anticoagulation modality during treatment^120^. These findings underscore the need for tailored anticoagulation and particular consideration of renal function in management strategies.

Direct oral anticoagulants (DOACs) also play a crucial role in managing patients with coronary disease and atrial fibrillation (AF) undergoing PCI. Antithrombotic therapy is essential for preventing stent thrombosis and reducing thromboembolic complications post-PCI. Current guidelines, including the 2017 European Dual Antiplatelet Therapy (DAPT) guidelines and 2018 U.S. expert recommendations, suggest short-term triple therapy following drug-eluting stent PCI and then transitioning to oral anticoagulants after one year. DOACs are favored over VKAs due to their safety profile and ease of use, as supported by randomized trials demonstrating a significant reduction in bleeding events such as transfusion-requiring bleeding, intracranial hemorrhage, and gastrointestinal bleeding in DOAC users^121^.

Prior to an elective PCI procedure, the AHA advises that DOACs be temporarily discontinued to minimize bleeding risks, particularly for patients with stable coronary disease and low thrombotic risk. Procedures can typically be performed 24 h after stopping DOACs like rivaroxaban, apixaban, and edoxaban. However, adjustments are necessary for renal function, particularly for dabigatran, which may require a discontinuation up to 72 h prior to PCI if renal function is impaired. In cases of major bleeding, reversal agents such as andexanet and idarucizumab are recommended as first-line treatments to counteract DOAC effects. If a patient’s specific anticoagulant is unknown, prothrombin complex concentrates may be used as an alternative to rapidly restore hemostasis and reduce mortality risk. Proper peri-procedural management and tailored dosing regimens can further mitigate bleeding risks in PCI patients receiving DOACs^122^.

In-hospital bleeding (IHB) is not merely an acute procedural complication – it carries substantial long-term consequences that must be considered when tailoring antithrombotic strategies. Spadafora et al. analyzed over 23,000 patients from the international PRAISE registry and found that patients who experienced IHB during hospitalization for ACS had significantly higher all-cause mortality, major bleeding, and reinfarction at 1-year follow-up compared to those without bleeding events^123^. Notably, patients with IHB were older, more likely to be female, and had more comorbidities, and were also less likely to be discharged on optimal medical therapy, highlighting how bleeding can prompt premature de-escalation of antithrombotic regimens. These findings underscore that early bleeding events should be viewed as a marker of frailty and a trigger for intensified long-term follow-up. Incorporating bleeding risk into procedural and pharmacological planning is therefore essential to optimize both immediate and downstream outcomes in ACS and PCI populations.

Bivalirudin, a direct thrombin inhibitor, has been studied extensively for its role in reducing bleeding in patients undergoing PCI, particularly compared to heparin (UFH or LMWH). Earlier studies, such as the MATRIX (Radial versus femoral access and bivalirudin versus UFH in invasively managed patients with ACS) trial, demonstrated that bivalirudin significantly reduced major bleeding and mortality when compared to heparin, especially when used in conjunction with GPIs. However, with the decline in routine GPI use and the adoption of modern PCI techniques (e.g., radial access), studies like the HEAT-PPCI (UFH versus bivalirudin in primary PCI) and VALIDATE-SWEDEHEART (Bivalirudin versus Heparin in ST-Segment and Non–ST-Segment Elevation Myocardial Infarction in Patients on Modern Antiplatelet Therapy in the Swedish Web System for Enhancement and Development of Evidence-based Care in Heart Disease Evaluated according to Recommended Therapies Registry Trial) trials found no clear advantage of bivalirudin over heparin, particularly when GPIs were only used provisionally^124^.

A recent meta-analysis supported these findings, showing that bivalirudin, regardless of whether a post-PCI bivalirudin infusion was used, did not significantly reduce 30-day all-cause or cardiac mortality, stent thrombosis, or major adverse cardiovascular and cerebrovascular events when compared to heparin. However, it was associated with fewer serious bleeding events, both at the vascular access and non-access sites, and it was shown to reduce rates of blood transfusions. The reduction in serious bleeding events was particularly notable when comparing bivalirudin to heparin with planned GPI use; even without planned GPI use, bivalirudin still showed a modestly lower bleeding risk when compared to heparin^125^. While the bleeding risk reduction benefits of bivalirudin are clear, its overall clinical advantage remains modest, and a high-dose post-PCI infusion may increase stroke risk. These findings suggest that bivalirudin’s role in PCI remains valuable for bleeding risk management, but its superiority to heparin is limited in broader settings.

### Stent selection

Stent selection is another critical decision-point for precision PCI, as choosing the most appropriate stent type and design tailored to the patient’s specific anatomical and clinical characteristics is essential for optimizing outcomes and minimizing complications. Modern drug-eluting stents (DES) consist of three key components: the stent scaffold, an antiproliferative drug, and a drug-elution mechanism. Originally made with thick-strut stainless steel, stent scaffolds now incorporate thinner struts and advanced alloys like cobalt-chromium, which improve flexibility, strength, and deliverability^126^. Most DES platforms use sirolimus-based drugs, which are safer and more effective in reducing restenosis and stent thrombosis than earlier paclitaxel-based designs^127^. Innovations in drug-eluting mechanisms, including bioabsorbable and abluminal-only polymers, have further enhanced safety by reducing risk of late complications. Some stents, such as BioFreedom, even eliminate polymers altogether, applying drugs directly to the stent surface^128^. These advancements have established DES as the standard treatment for most PCI cases, improving outcomes across a diverse array of patient populations.

When selecting a stent, several factors must be considered. The duration of DAPT is particularly important for patients at high bleeding risk. Newer DES like BioFreedom have demonstrated safety with only one month of DAPT, making bare-metal stents largely obsolete in such cases. Other factors include stent size and expansion capacity, which are critical in complex cases, such as left main bifurcations, to prevent issues like incomplete strut apposition. Deliverability is another key factor to consider, as modern thin-strut DES are easier to deliver than their predecessors. It is also important to note that stent designs which feature fewer connectors between stent rings offer improved flexibility and deliverability, while others may be prone to deformation or fracture^126^.

Strut thickness also has a significant impact on clinical outcomes. A study by Bangalore et al. demonstrated that ultrathin-strut stents, considered third-generation DES, are associated with a 16% reduction in target lesion failure (TLF) and lower rates of stent thrombosis compared to thicker-strut DES^128^. A meta-analysis by Saito et al. expanded on these findings, showing that thinner struts reduce early adverse events like target lesion revascularization (TLR) and stent thrombosis, although long-term outcomes (e.g., late and very late stent thrombosis) were not significantly affected^129^. Nevertheless, ultrathin stents come with limitations, including reduced fluoroscopic visibility, especially in labs without stent boost imaging, and potential loss of radial strength that may increase risks of lesion recoil or malapposition. This is especially relevant in heavily calcified or ostial lesions, although the clinical impact is generally limited if appropriate lesion preparation is performed^130^. Iglesias et al. found no difference in long-term outcomes between ultrathin (60 μm) biodegradable polymer sirolimus-eluting stent (BP-SES) and thin-strut (81 μm) durable polymer everolimus-eluting stent (DP-EES) in small vessel disease. This suggests that strut thickness may be less impactful in certain patient subsets^131^. These findings highlight the clinical importance of choosing stents with thinner struts to reduce adverse events and promote faster endothelialization, minimizing risks of restenosis and thrombosis^132^.

Intuitively, certain stent platforms are better suited for specific anatomical challenges. For example, patients who are at high-risk for bleeding complications benefit from the BioFreedom stent, which requires only four weeks of DAPT and has demonstrated superior safety compared to bare-metal stents. Ostial coronary lesions may require stents with better longitudinal strength, while tortuous vessels are best treated with highly flexible and fracture-resistant designs. Patient and target lesion characteristics should guide stent selection for these reasons^126^.

Additionally, there are a number of specific lesion subsets that have good evidence for stenting with second-generation DES. Generally, these subsets are considered higher risk of adverse outcomes and include lesions such as aorto-ostial, left main coronary, bifurcation, CTO, in-stent restenotic, or long lesions. In the case for aorto-ostial lesions, several studies have demonstrated newer-generation DES to have a low incidences of TLF, ISR and TLR at 3 years follow^133,134^. For management of left main lesions, Everolimus-eluting stents (EES) were shown to be noninferior to coronary artery bypass grafting (CABG) among a cohort with low or intermediate SYNTAX scores, when assessing a composite endpoint of death, stroke, or MI at 3 years follow up^135^. Of the remainder of these complex, high risk lesions, newer-generation DES are often implemented to ensure adequate coverage and reduction in the risk of restenosis. EES were demonstrated to decrease incidence of in-segment restenosis (11.8% vs 31.4%) and MACE (8.9% vs 22.6%) compared to paclitaxel-eluting stents (PES)^136^. Burzotta et al. compared sirolimus-eluting stents (SES) and everolimus-eluting stents (EES) in bifurcation lesions treated by provisional stenting and found that EES provided better results in the side branch without significant differences in major adverse events^137^.

Looking forward, bioresorbable stents are viewed as the future of PCI. Despite promising early results, currently, bioresorbable stents face challenges like lower radial strength, poor deliverability, and limited overexpansion capacity. Their clinical use remains limited to simple lesions due to these drawbacks, and their long-term benefits are yet to be fully proven^126^. Future iterations with thinner struts may expand their application to more complex lesions. Until then, meticulous lesion preparation and frequent imaging are necessary for successful use of these devices. Continued research and development are essential to determine whether bioresorbable stents will fulfill their theoretical advantages over permanent metallic implants.

### Complex strategies: Bifurcation Lesions, Chronic Total Occlusion (CTO), and Left Main Lesions

#### Bifurcation Lesions

Bifurcation lesions are often classified based on presence of stenosis prior to intervention with the Medina classification. This classification system is denoted as three binary digits for each respective segment of the bifurcation, the proximal main vessel, the distal main vessel, and the side branch. Each digit represents presence (1) or absence (0) of stenosis^138^. This systematic method helps guide the best approach to intervention of these lesions, generally involving provisional or two-stent strategies. For more complex lesions, such as those with significant side branch involvement (e.g., Medina 1,1,1 or 0,1,1), two-stent strategies may be considered. Techniques such as double kissing crush (DK-crush), mini-crush, or culotte stenting are often employed in these scenarios, depending on the specific anatomical and morphological characteristics of the lesion^139,140^. Stent selection in these circumstances also becomes significant. In addition to second-generation stent placement for the management of bifurcation lesions, dedicated bifurcation stents, such as the Axxess and Biomatrix, have been proposed as alternatives to dual-stent techniques. The COBRA trial showed that a dedicated bifurcation stent strategy resulted in similar stent strut coverage at 9 months with good clinical outcomes at 5 years compared to the culotte stenting technique using Xience stents^141^. Additionally, the study by Gasior et al. demonstrated that the bifurcation-dedicated DES (Bioss LIM C) had favorable biomechanical properties and lower thrombogenicity at the side branch ostia compared to conventional DES^142,143^. Overall, second-generation DES should be selected for management of bifurcation lesions. Provisional stenting remains the preferred strategy for most bifurcation lesions, but dedicated bifurcation stents can be considered in cases in true bifurcation lesions with side branches greater than 2.25 mm in size, cases with complex anatomy or where the main and side branch have significant disease, or finally if the operator deems there to be high risk of side branch compromise^139,141,142,144–146^.

#### Chronic Total Occlusion (CTO)

Similarly, management of CTOs should generally involve second-generation DES placement as supported by the EXPERT trial which demonstrated favorable procedural success and lower composite adverse events at one year follow up^147^. However, complexity of CTO lesions requires thoughtful pre- and intra-procedural considerations. Multiple scoring systems are available to predict successful intervention on CTO lesions including the J-CTO score, used to predict success of crossing a CTO within 30 min. This score accounts for five independent factors, each contributing a single point to the collective score if present, including blunt stump appearance of the proximal cap of the occlusion, presence of a greater than 45-degree bend within the lesion, occlusion equal to or greater than 20 mm in length, presence of calcification within the CTO, and previous attempt and failure of intervention on the lesion^148^. An alternative is the Prospective Global Registry for the Study of Chronic Total Occlusion Intervention (PROGRESS-CTO) score which takes into account lesion cap ambiguity, moderate/severe tortuosity of the vessel, circumflex artery CTO, and the absences of collaterals^149^. In each of these scoring systems, higher scores are associated with lower success. Based on the complexity of the CTO, the interventional approach is determined, generally weighing four options: antegrade wire escalation (AWE), antegrade dissection and reentry (ADR), and retrograde wire escalation (RWE), and retrograde dissection and reentry (RDR)^150^. AWE is typically the first-line approach for less complex lesions and is often preferred when the proximal cap is well defined, the distal vessel is of good quality, or the occlusion is relatively short in length (<20 mm). ADR is an alternative that can be considered when AWE fails, or in cases where the lesions is long, tortuous, or heavily calcified. Alternatively, a retrograde approach is often implemented in cases where the proximal cap is ambiguous, the lesion lacks adequate collaterals, or an antegrade approach has failed^151^. RWE is preferred to RDR when there are suitable collaterals that would allow for controlled and precise wire passage – these lesions tend to not be long and lack heavy calcification. RDR is considered in the most complex CTO cases. While it is associated with higher technical success in these complex lesions, it also carries higher risk of complications such as coronary perforation^152^. In cases for which both an antegrade and retrograde approach have failed or deemed unsuitable, controlled antegrade and retrograde tracking (CART) can be implemented. Currently CART is the preferred retrograde wire-crossing technique used by most interventionalists managing CTOs^153^. Overall, CTO PCI should be considered in patients who would likely benefit from symptomatic and quality of life improvement, as definitive clinical outcomes have yet to be elucidated in clinical trials. A number of strategies may be implemented to cross these lesions – decisions regarding optimal approach should be guided by patient and lesions characteristics.

#### Unprotected Left Main Disease

UPLMD refers to significant stenosis (≥50% diameter stenosis) of the left main coronary artery without prior coronary artery bypass grafting (CABG) protection. It is a high-risk subset of CAD with the strongest evidence that revascularization provides survival benefit over medical treatment alone in stable patients^154^. Generally, CABG is recommended as the preferred revascularization strategy for UPLMD as it has been associated with improved survival when compared to PCI, and ad hoc PCI of UPLMD is discouraged^155^. However, PCI with DES is a reasonable alternative in select patient populations, namely those with favorable anatomy as defined by a low SYNTAX score (0–22) and high surgical risk^156^. In the cath lab, accurate assessment of UPLMD is essential. For all patients undergoing coronary stent implantation for UPLMD, intravascular imaging is recommended per the 2021 ACC/AHA/SCAI guidelines (Class IIa, LOE B) to further characterize the degree of calcium, volume and extent of plaque, as well as vessel size^157^. A recent study by Zhang et al. identified four independent PCI technological advances that that significantly influence 3-Year MACEs of UPLMD PCI, including postdilatation with noncompliant balloons at high pressure, implantation of second-generation DES, use of IVUS, and utilization of final kissing balloon inflation (FKBI)^158^. Most of the data for FKBI is limited to its use in management of bifurcation lesions, and this data is largely discordant suggesting that FKBI after 1-stent technique is either beneficial^159^, neutral^160^,or harmful^161^. Further, the EXCEL trial suggested that performance of FKBI after PCI for left main bifurcation lesions did not improve clinical outcomes^162^. There is good evidence for the implementation of the DK-crush technique in management of distal bifurcation lesions when managing left main disease. Evidence from the DKCRUSH-V trial demonstrated that the DK-crush technique is superior to provisional stenting (PS) for true distal left main bifurcation lesions. At one year, the DK-crush technique resulted in a significantly lower rate of target lesion failure (TLF) compared to PS (5.0% vs. 10.7%, *p* = 0.02)^163^. This benefit was primarily driven by reductions in target vessel myocardial infarction and stent thrombosis. Further supporting evidence from the DKCRUSH-V trial showed that the DK-crush technique continued to outperform PS at 3-years follow up, with lower rates of TLF (8.3% vs. 16.9%, *p* = 0.005), target vessel myocardial infarction, and target lesion revascularization^164^. In patients for which the optimal treatment strategy is unclear, a heart team approach that includes representation from interventional cardiology, cardiac surgery, and clinical cardiology is a Class I recommendation by both ACC/AHA/SCAI and ESC/EACTS to improve clinical outcomes^165^. When PCI is pursued, technical proficiency with imaging, bifurcation stenting strategies, and plaque modification is essential. The patient’s anatomy, comorbidities, and preferences, along with procedural risk and long-term benefit, should guide decision-making.

## Post-PCI care

### Hemostasis

TRA is a widely preferred approach for coronary angiography as it offers quicker recovery and lower vascular complications, particularly bleeding, when compared to transfemoral access^166^. However, a key drawback of TRA is RAO, which has been reported to occur in approximately 8% of patients. In an effort to combat this complication, the combination of a shorter duration of the radial hemostatic device (RHD), also known as the TR band, and patent hemostasis, where light pressure is applied to prevent bleeding without occluding the artery, has been shown to reduce the incidence of RAO^167^.

The TR Band protocol involves application following catheterization or PCI after arterial sheath removal. Initially, the band is inflated with 15 mL of air, though an additional 1–3 mL more may be added, if needed, to achieve hemostasis. It is important to note that the total air volume should not exceed 18 mL as this could compromise radial arterial blood flow. Once hemostasis is achieved, air is gradually removed starting at 90 min in 2-4 mL increments every 20 min, with continuous monitoring for bleeding. If bleeding occurs, 2 mL of air is reinflated, and the process restarts after 30 min of observation. In cases of hematoma development, additional air may be added to control bleeding^168,169^. Agents such as verapamil or nitroglycerin may be administered during this phase to prevent RAO by relaxing the artery, improving blood flow, and reducing complications^27^.

Studies have shown that RAO risk is reduced with smaller sheath sizes, adequate intra-procedural anticoagulation, and the use of patent hemostasis, although there is no consensus regarding the optimal duration of hemostasis^170^. The AHA position statement on TRA does not specify a recommended duration for hemostasis but notes that most physicians advise radial artery compression for at least 60 min after diagnostic procedures and between 120 and 180 min after PCI^27^.

A meta-analysis by Maqsood et al. aimed to evaluate the efficacy and safety of various TRA hemostatic banding durations compared to the standard two-hour duration in preventing RAO, access site hematoma, and rebleeding. This study found that shorter durations (<90 min) increased the risk of access site hematoma without significantly affecting RAO or rebleeding. Cluster plots depicting combined efficacy and safety outcomes indicated that a two-hour duration provided the optimal balance between efficacy (RAO prevention) and safety (reduced hematoma and rebleeding)^170^.

Additionally, the PRACTICAL-2 (Postcath Radial Arterial Clamp Time in the Cath Lab) trial examined whether shorter RHD durations could lower the risk of RAO in patients undergoing diagnostic cardiac catheterization via the TRA, using small sheaths (5 French) without adjunctive heparin. Patients were randomized to RHD durations of ten, twenty, or thirty minutes, followed by gradual release over twenty minutes. The primary outcomes were hematoma size and incidence of RAO one hour after device removal. Results indicated low hematoma rates, with only one patient developing a ≥ 5 cm hematoma in the twenty-minute group. RAO rates were 6.7%, 10.7%, and 6% in the ten, twenty, and thirty-minute groups, respectively, with no significant differences across duration groups. Clinically, this suggests that shorter RHD durations (ten or twenty minutes) do not significantly reduce RAO or hematoma risks, and that a thirty-minute duration strikes a safe balance for minimizing complications without unnecessary prolongation^167^.

### Post-PCI discharge

Traditionally, uncomplicated, elective PCI has been followed by overnight inpatient observation due to concern for potential peri-procedural adverse events, including acute vessel occlusion, MI, and vascular access complications. However, advancements in contemporary percutaneous techniques and anticoagulation strategies have significantly reduced the risk of major adverse events, prompting debate and the recent establishment of guidelines regarding same-day discharge (SDD) after PCI. The 2021 ACC Expert Consensus Decision Pathway on SDD emphasizes its feasibility and benefits for both patients and healthcare facilities^171^.

Contrary to common misconceptions, studies show that a significant majority of patients prefer SDD, valuing the opportunity to return home after their procedure. A recent prospective study of patients undergoing PCI for CTO revealed that the hospital readmission rate was low for both SDD and non-SDD patients^172^. Additionally, a 30-day follow-up indicated that those discharged on the same day expressed greater satisfaction with their discharge timing compared to patients discharged the next day, with a notable preference for SDD for future procedures^173^.

There is compelling evidence supporting not only patient satisfaction associated with SDD, but also safety of this practice. Two meta-analyses showed no significant differences in major adverse cardiovascular events (MACE) when comparing same-day and next-day discharge following PCI^174,175^. Moreover, it has been well-demonstrated that the inpatient environment can hinder recovery due to a lack of privacy and frequent nighttime disruptions, potentially leaving patients vulnerable to posthospitalization syndromes^176^.

Implementing SDD after PCI can also alleviate pressure on healthcare facilities by optimizing patient flow and reducing demand for inpatient beds, leading to greater potential savings for U.S. healthcare systems annually^171^. This suggests that adopting SDD not only enhances the overall efficiency of healthcare delivery, but also improves patient experiences, satisfaction, and outcomes.

Follow-up remains an important aspect of post-PCI care. The Society for Cardiovascular Angiography & Interventions (SCAI) recommends follow-up within four weeks of discharge and earlier for patients with renal insufficiency, anemia or documented procedural complications^3^. Routine functional testing is not generally recommended. Surveillance with imaging-based stressed testing at six (Class IIb recommendation) and twelve (Class IIb recommendation) months post-PCI should be considered in high-risk patient populations; though the POST-PCI (Pragmatic Trial Comparing Symptom-Oriented versus Routine Stress Testing in High-Risk Patients Undergoing PCI) trial found no significant differences in major cardiovascular events or mortality at two years follow-up when comparing routine functional testing versus standard of care in high-risk patients^177,178^.

### Genetic testing for P2Y12 inhibitors post-PCI

DAPT, which combines aspirin with a P2Y12 inhibitor, is the standard treatment to reduce ischemic events for patients undergoing DES PCI. However, a “one-size-fits-all” approach to DAPT can lead to suboptimal outcomes, especially when prescribing clopidogrel, which may be less effective in certain patients due to genetic variability in metabolism of clopidogrel, a prodrug, to its pharmacologically active form^179^. Approximately 30% of European, 35% of African, and 60% of East Asian ancestry individuals have a *CYP2C19* loss-of-function allele that results in reduced clopidogrel bioactivation^180^. A recent AHA statement asserts that the evidence supports *CYP2C19* genetic testing to guide the selection of P2Y12 inhibitors, particularly in patients with ACS^181^. This evidence includes studies showing that carriers of a *CYP2C19* loss-of-function allele experience reduced clopidogrel activation and higher platelet reactivity placing them at higher risk of MACE^182,183^. Additional data demonstrate that selection of P2Y12 inhibitor therapy based on *CYP2C19* genotype improves clinical outcomes^184,185^.

Two large clinical trials of genotype-guided DAPT, TAILOR-PCI (Tailored Antiplatelet Initiation to Lessen Outcomes due to Decreased Clopidogrel Response After PCI) and POPular Genetics (Cost-effectiveness of CYP2C19 Genotype Guided Treatment With Antiplatelet Drugs in Patients With ST-segment-elevation Myocardial Infarction Undergoing Immediate PCI With Stent Implantation: Optimization of Treatment), have been conducted. The POPular Genetics trial demonstrated that a genotype-guided approach, where ticagrelor or prasugrel is prescribed to *CYP2C19* loss-of-function allele carriers and clopidogrel was prescribed to noncarriers, reduced the risk for bleeding compared to universal use of ticagrelor or prasugrel without compromising risk for ischemic events^185,186^. The TAILOR-PCI trial showed a trend towards reduced ischemic events with genotype-guided therapy versus universal clopidogrel use, although it did not reach statistical significance for the primary composite endpoint of cardiovascular death, myocardial infarction, stroke, stent thrombosis, and recurrent ischemia^186^. However, a pre-specified analysis of cumulative ischemic events in the same trial indicated a significant reduction in risk in the genotype-guided group^187^. Multi-site observation studies and meta-analyses have shown reduced risk of ischemic events with prasugrel or ticagrelor versus clopidogrel in loss-of-function allele carriers, but no difference in ischemic events with alternative therapy versus clopidogrel in non-carriers^184,186,188,189^.

The 2016 guidelines from the American College of Cardiology (ACC) and the AHA do not recommend routine genetic testing for all patients but do acknowledge its potential benefit in selected cases^190^. The 2021 and 2025 guidelines from the ACC/AHA/Society for Cardiovascular Angiography and Intervention do not address data in support of genetic testing or provide recommendations related to genotyping to guide antiplatelet therapy^157^. According to a recently updated international consensus statement, it is reasonable to consider genotyping (or platelet function testing) to guide P2Y12 inhibitor selection following ACS and PCI in patients at high bleeding and ischemic risk, in whom clopidogrel can be used in place of prasugrel or ticagrelor in those without a loss-of-function allele^191^. In practice, genetic testing should not be used in isolation; rather, it must be considered alongside other key factors, such as clinical risk, angiographic complexity, and socioeconomic considerations. While genetic testing shows promise for personalizing antiplatelet therapy, its use is currently confined to a limited number of centers rather than being a standard approach for all patients^192^.

## Future directions

The future of precision-based PCI is being shaped by the development and integration of innovative devices that hold promise for addressing complex coronary pathologies and optimizing outcomes in select patient populations through individualized care approaches.

Among these innovations is the use of coronary sinus occluders (CSO) for refractory angina in patients who are not candidates for revascularization. The CSO is a balloon-expandable device that creates focal narrowing within the CS and subsequently increases pressure and redistributes blood flow to the ischemic myocardium. Evidence supporting this approach is limited but growing. The REDUCER-I (An Observational Study of the Neovasc Reducer™ System) study reported a 99% procedural success rate among patients treated with the CS Reducer, with improvements in angina severity and reduction in Canadian Cardiovascular Society (CCS) class scores at two years – 82% showed improvement of at least one CCS class^193^. In the COSIRA (Coronary Sinus Reducer for Treatment of Refractory Angina) trial, 35% of patients who received a CSO experienced an improvement of at least two CCS classes at six months, with a significant improvement in quality of life measures^194^. The RESOURCE (Reducer Efficacy and Safety from an internatiOnal mUlticenteR Clinical rEgistry) study similarly found that 39.7% of patients improved by at least two CCS classes at a median follow-up of 502 days, with demonstration of a high procedural success rate of 96.7% and low complication rate^195^. While these findings are encouraging, the overall evidence is limited by small study sizes and observational designs, and the clinical benefit of CSOs remains preliminary. Advancements in imaging modalities, such as CMR and quantitative flow ratio, may further aid in the selection and monitoring of patients, ensuring more precise use of this emerging technology.

Another promising innovation in the realm of PCI is the application of drug-coated balloons (DCBs), which play a significant role in treating CAD and have already become a mainstay for CAD treatment in many parts of the world, particularly for ISR, small-vessel disease, or bifurcation lesions. DCBs deliver antiproliferative agents, such as paclitaxel, directly to the vessel wall in an effort to reduce neointimal hyperplasia without implementing a permanent implant^196^. For de novo lesions, however, the evidence is less clear. The BASKET-SMALL 2 (The Basel Stent Kosten Effektivitäts Trial Drug Eluting Balloons vs Drug Eluting Stents in Small Vessel Interventions) trial demonstrated that DCBs are non-inferior to DES in small vessel disease, with similar rates of MACE at 12 months^197^. Additionally, the DEBUT (The Drug-Eluting Balloon in Stable and Unstable Angina) trial showed that DCBs are superior to bare-metal stents in patients with high bleeding risk, suggesting a potential benefit in specific patient populations^198^. DCBs are particularly advantageous in scenarios where avoiding a permanent stent is desirable, such as in small vessels, bifurcation lesions, and patients with high bleeding risk, as they circumvent the need for prolonged DAPT^199^. However, several limitations should be considered. DCBs are associated with higher costs compared to conventional approaches, and there is potential for overuse, particularly in settings where operators may be incentivized to treat small branch lesions that could otherwise be managed medically. Careful patient selection and adherence to evidence-based indications are essential to optimize outcomes and minimize unnecessary interventions. Further studies are needed to confirm their broader applications and validate their safety and efficacy in various clinical settings. Unlike DES, DCBs deliver antiproliferative drugs directly to the vessel wall without leaving behind a permanent scaffold, reducing the risk of late complications such as stent thrombosis. The individualized application of DCBs in PCI depends on detailed lesion assessment, including vessel size, plaque morphology, and previous stent history. Precision strategies leveraging intravascular imaging techniques such as OCT and IVUS can enhance patient selection and procedural planning for DCB use. In parallel, ongoing developments in DCB drug formulations and delivery systems are expected to expand their applications, improving efficacy and reducing the need for repeated interventions in the years to come.

Among emerging stent technologies, the DynamX sirolimus-eluting coronary bioadaptor system exemplifies a next-generation approach to precision PCI. Unlike conventional DES, which create a permanent rigid metallic cage, DynamX incorporates helical cobalt-chromium strands coated with a bioresorbable polymer. After implantation, the polymer degrades in vivo, allowing the helical strands to unlock and separate, which restores vessel motion and enables adaptive remodeling. This design aims to maintain acute procedural performance comparable to second-generation DES while permitting the vessel to regain physiological function and cyclic pulsatility, potentially reducing long-term complications such as target lesion failure and persistent angina^200,201^. Clinical imaging studies demonstrate that DynamX maintains lumen area while allowing for positive vessel and device area remodeling, as well as restoring cyclic changes in lumen diameter during the cardiac cycle. These properties may translate into improved long-term outcomes, including reduced need for repeat revascularization and enhanced symptom relief. Ongoing randomized trials, such as INFINITY-SWEDEHEART, are evaluating its safety and efficacy relative to contemporary DES platforms across broad PCI populations^200^. The integration of DynamX into precision PCI highlights a broader paradigm shift toward devices that not only treat the target lesion but also preserve or restore vessel physiology, complementing strategies such as DCBs and CSOs, and further individualizing therapy based on patient- and lesion-specific characteristics.

Despite these device-level advances, logistical and operational precision remains an underdeveloped area in PCI. One key example is the lack of automated or machine learning-based tools to guide decisions around same-day discharge. While protocols exist for identifying low-risk patients who may be safely discharged shortly after PCI, these decisions are often subjective and vary center to center. Future development of predictive algorithms that integrate patient-specific variables, procedural factors, and real-time hemodynamic data could standardize and optimize discharge planning–offering another layer of precision with potential to reduce costs and improve patient satisfaction.

While the promise of precision-based PCI is compelling, its widespread adoption in routine clinical practice faces significant challenges. Chief among these are cost, scalability, and variability in access to advanced imaging modalities and specialized technologies across institutions. Devices such as intravascular imaging tools, DCBs, CSOs, and left ventricular support devices often carry higher upfront costs compared with conventional PCI strategies. In many healthcare systems, these tools are either not reimbursed or only partially reimbursed, creating financial disincentives for operators and institutions. If all of the pre-, intra-, and post-procedural elements of a fully realized precision PCI strategy were applied in a single case, including multimodality intravascular imaging, physiologic assessment, specialized devices, advanced pharmacology, and digital support platforms, the procedural cost could quickly become prohibitive. Moreover, implementing individualized procedural strategies demands time, multidisciplinary coordination, and data integration infrastructure that may not be readily available in all cath labs. Though, the financial challenge extends beyond individual procedures, as widespread implementation would require costly investments in equipment, software, infrastructure, and training. These barriers risk widening disparities, with well-funded centers able to adopt precision strategies more readily than resource-limited institutions.

Looking forward, future directions must include cost-effectiveness research to identify which precision components deliver the greatest incremental benefit, alongside reimbursement reforms, scalable adoption strategies, and development of lower-cost technologies. Integration of innovative devices such as CSOs, DCBs, and DynamX bioadaptors, coupled with logistical optimization and evidence-based precision frameworks, may ultimately enable precision PCI to move from concept to practical, population-level implementation.

While these device- and procedure-level innovations advance precision PCI, their optimal implementation increasingly relies on digital capabilities to integrate patient data, guide real-time decision-making, and support complex interventions.

## Digital Capabilities in the Modern Cath Lab

Realizing the full potential of the aforementioned future precision PCI tools will require sophisticated digital infrastructure. Advanced imaging, extended reality platforms, and integrated data systems provide the means to individualize procedural planning, guide real-time decisions, and support complex interventions, complementing device- and procedure-level innovations.

The digital transformation of the cardiac catheterization lab is reshaping the delivery of coronary care, enabling earlier diagnosis, deeper pathophysiologic insights, and more efficient procedural workflows. One of the most promising frontiers is the ability to detect vulnerable plaque before it manifests as an acute coronary event. While IVUS and OCT remain the gold standard imaging modalities for identifying lesions that are prone to progress, they provide limited utility in detecting vulnerable plaques^202^. Novel diagnostic tools—such as near-infrared spectroscopy (NIRS), near-infrared autofluorescence (NIRAF), near-infrared fluorescence (NIRF), virtual histology IVUS, fluorescence lifetime imaging (FLIm), and intravascular photoacoustic imaging (IVPA)—have been developed and directly implemented into the angiographic workflow, to be used in parallel with IVUS or OCT such that clinicians can gain a more complete picture of CAD beyond simple luminal stenosis. Among these hybrid imaging modalities, NIRS IVU, OCT NIRS, OCT NIRF, and FLIm OCT are now available for clinical use, while FLIm IVUS^203^ and IVPA IVUS^204,205^ are still in preclinical development.

Equally important is the integration of data from multiple sources (imaging, physiologic measurements, historical ECGs, and stress test results) into a unified, interoperable platform. While there exist FDA-approved machine-learning systems dedicated to risk assessment such as eCART, APPRAISE-HRI, and the CLEWICU System, systems that support real-time data visualization and provide archival solutions that make prior studies easily accessible and transferable for multidisciplinary consultations intraprocedurally have yet to be developed. Streamlining this process avoids redundancy, enhances collaboration, and speeds up clinical decision-making.

Advanced digital imaging and 3D reconstruction techniques are increasingly being incorporated to improve procedural precision, particularly in the setting of reconstruction of coronary bifurcation anatomy, allowing for clinician to detect flow-limiting lesions more reliably^85^. Development of three-dimensional reconstruction by integration of angiography with OCT has also been validated and demonstrated high accuracy and reproducibility, which has become an integral tool for planning and optimizing intervention strategies^206^. These technologies can render detailed, patient-specific coronary anatomy and facilitate intelligent navigation within the coronary vasculature.

VR simulations are increasingly being used in PCI to enhance both planning and operator performance. These platforms offer highly immersive and interactive environments that use patient-specific data to model 3D intervention scenarios. This allows interventionalists to rehearse complex procedures in advance, improving familiarity with challenging anatomy. Studies have shown that cardiologists and trainees using VR-based simulation systems report improved user experience and enhanced procedural skill acquisition compared to traditional training methods^207^.

Beyond VR, augmented reality (AR) and mixed reality (MR) technologies enable the overlay of digital data, such as fluoroscopy or intravascular imaging, onto real-time procedural views, aiding intra-procedural navigation, stent placement, and spatial orientation^208,209^. Collectively, these tools fall under the umbrella of extended reality (XR), which is being integrated across all phases of cardiovascular care: pre-procedural planning, real-time procedural guidance, and even post-procedural debriefing^88,210^. In particularly complex scenarios like CTOs or anomalous coronary anatomies, XR systems support the selection of optimal catheters and access strategies while minimizing contrast and radiation exposure^45^.

To support this level of sophistication, cath labs must be staffed with dedicated information technology professionals who can manage the growing ecosystem of evolving hardware, software applications, and monitoring tools. These IT teams play a vital role in maintaining system integrity, eliminating data silos, and ensuring seamless interoperability across platforms, which in turn improves procedural efficiency and patient satisfaction. Moreover, as vascular interventions increasingly span multiple disciplines, including interventional cardiology, vascular surgery, and interventional radiology, a team-based, cross-specialty approach will be essential. The modern cardiovascular suite should be built with this collaboration in mind, physically and digitally equipped to accommodate complex, multimodal procedures.

Finally, cognitive and physical assessments should be integrated into data collection tools to create a more holistic understanding of each patient. Pulling structured data directly from the EMR, rather than requiring duplicate entry, can reduce clerical burden and create more accurate, consolidated datasets to guide clinical care and support future research.

## Conclusion

**Fig. 1:**
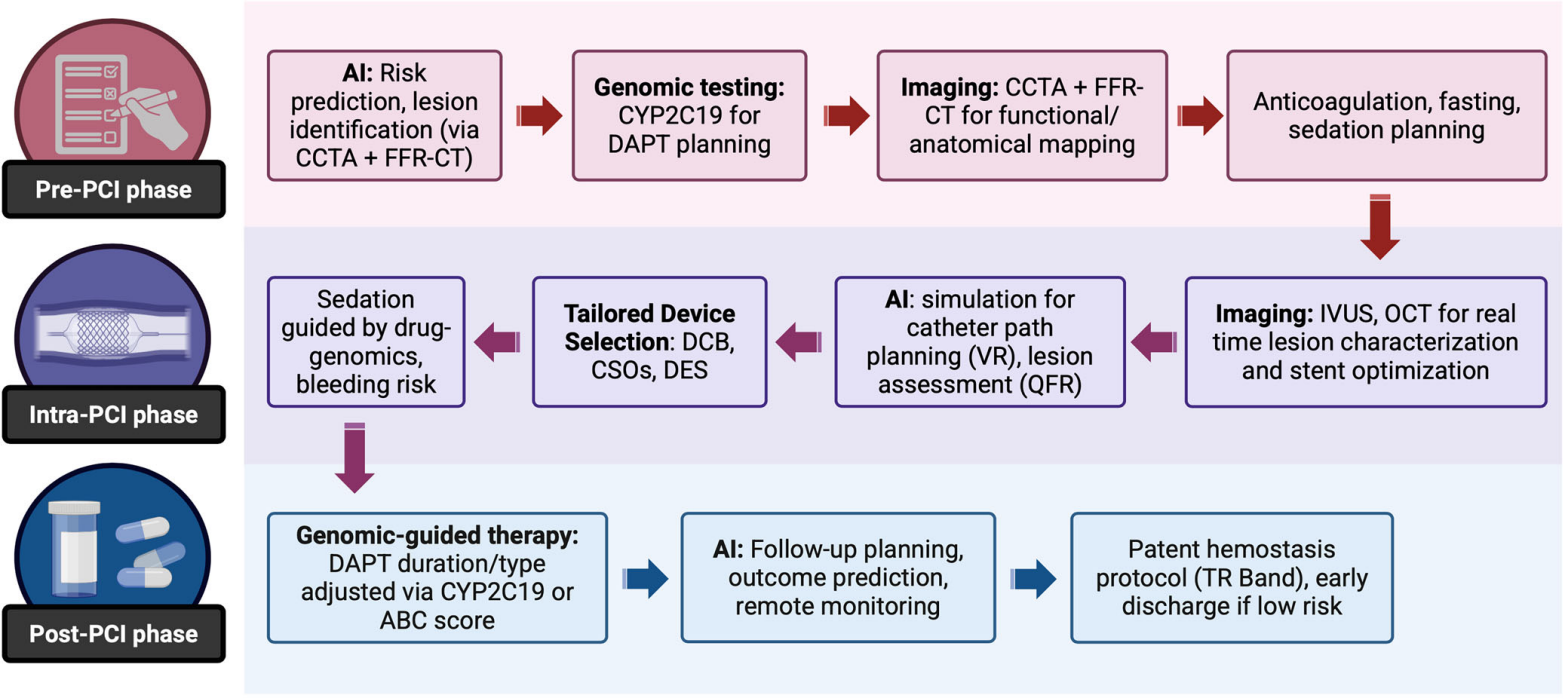
Tailored Approach to Precision Percutaneous Coronary Intervention and Procedural Management. A proposed step-by-step algorithm to incorporate precision medicine into pre-, intra- and post-PCI management of patients. (Figure created with BioRender.com)

**Fig. 2:**
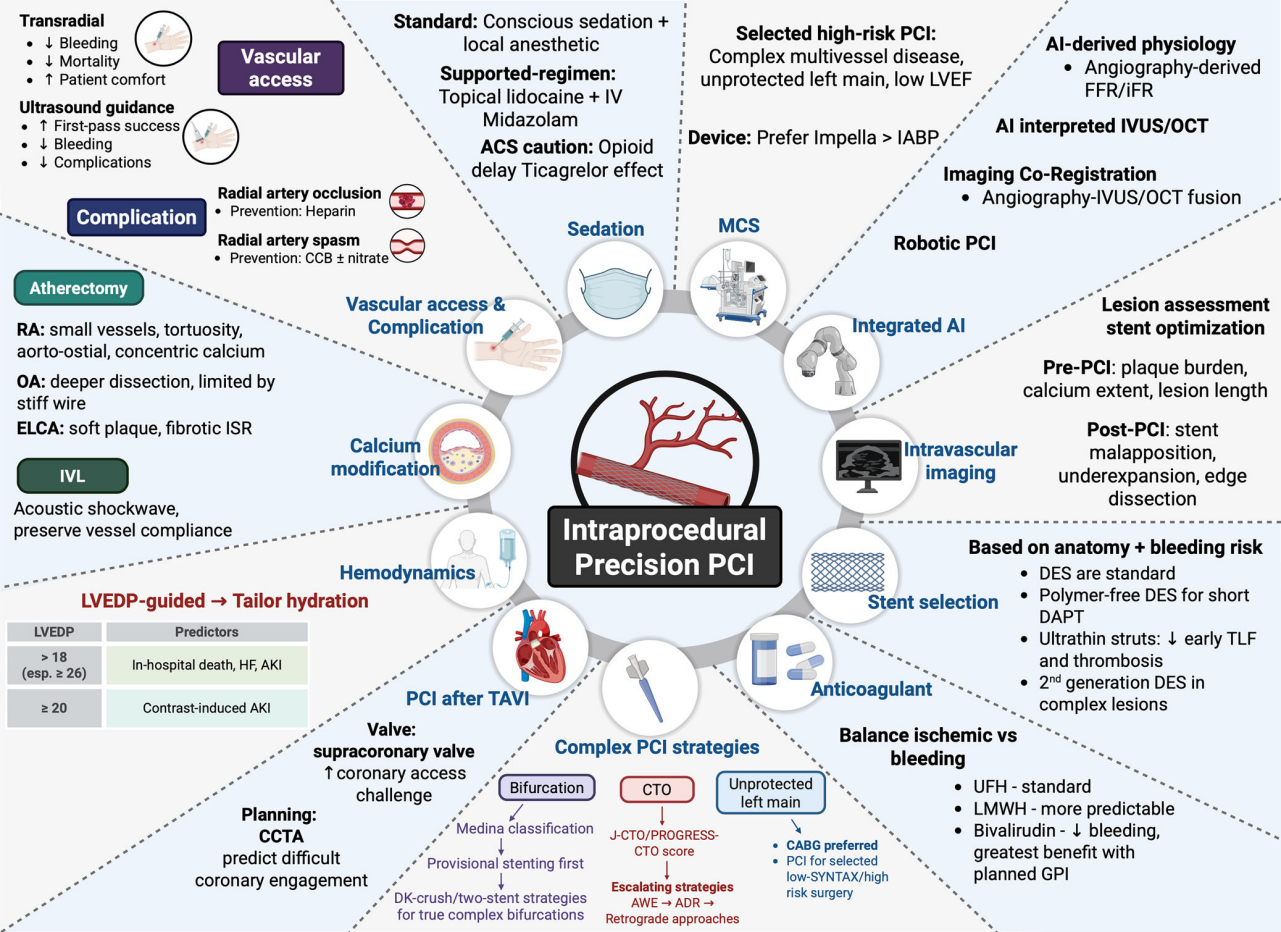
Intraprocedural precision percutaneous coronary intervention. This schematic illustrates a comprehensive framework for intraprocedural precision PCI, integrating patient-specific risk assessment, lesion complexity, and advanced technologies to optimize procedural safety and outcomes. (Figure created with BioRender.com)

**Fig. 3:**
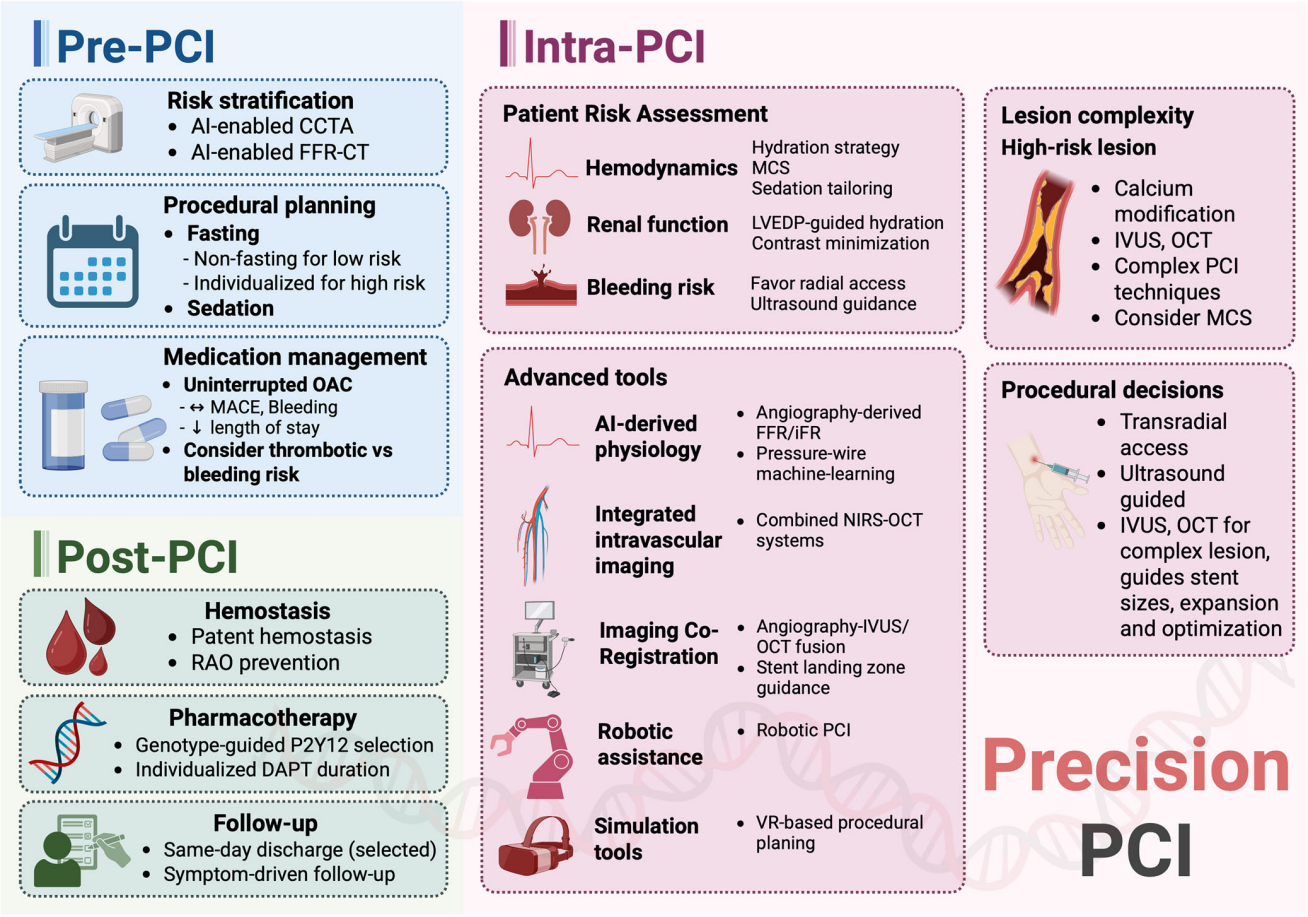
Precision percutaneous coronary intervention across the procedural continuum. This central illustration summarizes a precision-based PCI framework spanning pre-procedural, intraprocedural, and post-procedural phases. (Figure created with BioRender.com)

Precision PCI has the potential to redefine interventional cardiology over the next decade. By integrating intravascular imaging, physiologic assessment, digital tools, and tailored pharmacology and device strategies, precision PCI enhances diagnostic accuracy, procedural safety, and patient outcomes across the entire care continuum. We propose a tailored, pragmatic approach that utilizes precision medicine to guide pre-, intra-, and post-procedural management of patients undergoing PCI (Fig. 1). A comprehensive framework for intraprocedural precision PCI is illustrated in Figs. 2, 3. Additionally, the aforementioned advantages of precision PCI care over traditional PCI care are summarized in Table 1. Key characteristics of the randomized controlled trials are summarized in Table 2.

**Table 1:** Comparison of traditional vs. precision PCI for strategies, innovations, and limitations

Key Elements	Traditional PCI	Precision PCI	Advantages of Precision PCI
Patient Selection:	Angiography-guided decision-making	AI-based FFR-CT, CCTA, genomic risk scoring, clinical modeling	More accurate, individualized risk stratification
Imaging Guidance:	2D angiography only	Intravascular imaging (IVUS/OCT), virtual FFR, plaque morphology characterization	Optimized lesion prep, improved stent expansion, better outcomes
Pharmacology:	Empiric DAPT (e.g., clopidogrel for all)	CYP2C19-guided P2Y12 selection, LMWH or DOAC tailored to renal/genetic profiles	Reduced bleeding, better efficacy
Access Strategy:	Radial or femoral access based on anatomy/operator preference	Ultrasound-guided radial access, VR-based simulation for catheter path planning	Fewer complications, higher first-pass success
Device Selection:	Second-gen DES used universally	Tailored selection of DCBs, CSOs, or thin-strut DES based on anatomy, lesion complexity, and bleeding risk	Better device-lesion matching; lower restenosis in niche populations
Post-PCI Management	Standard DAPT duration, overnight admission	Genotype-adjusted DAPT, same-day discharge for low-risk patients	Higher satisfaction, lower costs, lower bleeding risk
Limitations	One-size-fits-all care; procedural variability	Requires advanced infrastructure, cost, clinician familiarity	Implementation barriers but high potential payoff

**Table 2:** Summary table of randomized controlled trials cited in the manuscript

Name of Trial	Population/Sample	Year	Importance
TONIC Trial (Boukantar et al.)	Patients undergoing interventional coronary procedures	2024	Examines safety of nonfasting vs fasting before coronary interventions; informs pre-procedure fasting guidelines
SCOFF Trial (Ferreira et al.)	Patients undergoing catheterization lab procedures	2023	Protocol for non-inferiority RCT on no fasting prior to cath; informs procedural safety and patient comfort
NXT Trial (Nørgaard et al.)	Patients with suspected coronary artery disease	2014	Evaluates diagnostic performance of noninvasive FFR derived from coronary CT angiography; informs noninvasive assessment of CAD
Woodhead et al.	Patients undergoing cardiac catheterization and PCI	2007	Investigates whether premedication increases vascular access complications; informs procedural medication safety
Elattar et al.	Patients undergoing radial coronary angiography	2023	Evaluates topical lidocaine and/or IV midazolam for prevention of radial artery spasm; informs strategies to reduce procedural complications
Deftereos et al.	Patients undergoing transradial coronary interventions	2013	Assesses moderate sedation and opioid analgesia for preventing radial artery spasm; informs sedation protocols to improve procedural outcomes
ATLANTIC Trial (Montalescot et al.)	STEMI patients receiving prehospital vs in-hospital ticagrelor	2014	Evaluates prehospital administration of ticagrelor in STEMI; informs timing of antiplatelet therapy for improved outcomes
PACIFY Trial (McEvoy et al.)	Patients undergoing PCI	2018	Assesses effect of IV fentanyl on ticagrelor absorption and platelet inhibition; informs peri-procedural opioid use and antiplatelet efficacy
MonAMI Trial (Saad et al.)	Acute MI patients receiving morphine ± metoclopramide	2020	Evaluates impact of morphine (with/without metoclopramide) on ticagrelor-induced platelet inhibition; informs opioid management in AMI and antiplatelet therapy timing
Access Study (Kiemeneij et al.)	Patients undergoing percutaneous transluminal coronary angioplasty	1997	Compares radial, brachial, and femoral approaches for PCI; informs choice of access site based on safety and efficacy
RADIAL-CABG Trial (Michael et al.)	Patients undergoing coronary artery bypass graft angiography and intervention	2013	Randomized comparison of transradial vs transfemoral access for CABG angiography and intervention; informs optimal access site selection in post-CABG patients
Verapamil RCT (Beyranvand et al.)	Patients undergoing coronary angiography	2022	Assesses effect of verapamil on reducing radial artery thrombosis; informs pharmacologic strategies to prevent radial artery complications
Lee et al.	Patients undergoing distal radial coronary angiography	2023	Compares spasmolytic regimens for preventing radial artery spasm during distal radial approach; informs optimal pharmacologic strategy to minimize procedural spasm
NISTRA Trial (Bouchahda et al.)	Patients undergoing transradial PCI	2023	Evaluates combination therapy with nicardipine and isosorbide dinitrate to prevent radial artery spasm; informs optimal pharmacologic prevention strategies
POSEIDON Trial (Brar et al.)	Patients undergoing coronary angiography at risk for contrast-induced AKI	2014	Tests hemodynamic-guided fluid administration to prevent contrast-induced acute kidney injury; informs peri-procedural fluid management strategies
Gu et al.	PCI patients at risk for contrast-induced nephropathy	2021	Uses LV end-diastolic pressure and BNP-guided low-dose furosemide to prevent contrast-induced nephropathy; informs individualized fluid/diuretic strategies
ACTIVATION Trial (Patterson et al.)	Patients undergoing TAVR with planned PCI	2021	Evaluates outcomes of performing PCI prior to transcatheter aortic valve implantation; informs timing and strategy of PCI in TAVR patients
PROTECT II Trial (O’Neill et al.)	Patients undergoing high-risk PCI requiring hemodynamic support	2012	Compares Impella 2.5 vs intra-aortic balloon pump for high-risk PCI; informs choice of mechanical circulatory support to optimize procedural outcomes
Protect III Trial (O’Neill et al.)	Patients with severely depressed LVEF undergoing PCI	2022	Demonstrates improved outcomes in high-risk patients with severely reduced LVEF using contemporary PCI practices; informs risk mitigation and procedural strategies
RENOVATE-COMPLEX-PCI Trial (Lee et al.)	Patients with complex coronary artery lesions undergoing PCI	2023	Demonstrates that intravascular imaging–guided PCI reduces target vessel failure compared to angiography-guided PCI; informs procedural optimization strategies
PREPARE-CALC Trial (Abdel-Wahab et al.)	Patients with severely calcified coronary lesions undergoing PCI	2018	Compares high-speed rotational atherectomy vs modified balloon angioplasty before drug-eluting stent implantation; informs optimal lesion preparation strategies
LOW-RAO Trial (Didagelos et al.)	Patients with radial artery occlusion post-transradial catheterization	2022	Investigates the efficacy of low-molecular-weight heparin in improving radial artery patency; informs treatment strategies for RAO post-procedure
CLARITY-TIMI 28 Trial (Sabatine et al.)	STEMI patients receiving fibrinolytics	2005	Compares low-molecular-weight heparin vs unfractionated heparin in STEMI patients treated with fibrinolytics; informs optimal anticoagulation strategy in STEMI
SYNERGY Trial (Ferguson et al.)	High-risk NSTEMI patients managed with early invasive strategy	2004	Compares enoxaparin vs unfractionated heparin in high-risk NSTEMI patients; informs optimal anticoagulation strategy for early invasive management
BIOSCIENCE Subgroup Analysis (Iglesias et al.)	Patients with small vessel coronary artery disease undergoing PCI	2019	Compares long-term outcomes of ultrathin-strut vs thin-strut DES; informs stent selection for small-vessel PCI to improve safety and efficacy
EXCEL Trial (Stone et al.)	Patients with left main coronary artery disease	2016	Compares outcomes of everolimus-eluting stents vs coronary artery bypass grafting for left main disease; informs revascularization strategy for LMCA disease
SEA-SIDE Trial (Burzotta et al.)	Patients with bifurcated coronary lesions undergoing PCI	2011	Prospective randomized comparison of sirolimus- vs everolimus-eluting stents using a provisional approach; informs stent selection and technique for bifurcation PCI
TRYTON Trial (Généreux et al.)	Patients with de novo true bifurcation lesions involving large side branches	2015	Compares dedicated bifurcation stent plus DES vs provisional SB balloon angioplasty plus DES; informs optimal strategy for complex bifurcation PCI
EXPERT CTO Trial (Kandzari et al.)	Patients with chronic total coronary occlusions undergoing PCI	2015	Evaluates safety and effectiveness of everolimus-eluting stents in CTO PCI; informs optimal stent selection and procedural strategies for complex chronic occlusions
PROGRESS-CTO Registry	Patients undergoing chronic total occlusion PCI	2012–2022	International registry analyzing outcomes of CTO PCI; informs procedural strategies, complication risk assessment, and scoring systems like PROGRESS-CTO and J-CTO
Nordic-Baltic Bifurcation Study III (Niemelä et al.)	Patients with coronary bifurcation lesions undergoing main vessel stenting	2011	Randomized comparison of final kissing balloon dilatation versus no kissing balloon; informs technique optimization for bifurcation PCI and reduction of side branch compromise
DKCRUSH-V (Zeng et al.)	Patients with left main distal bifurcation lesions	2017	Compares Double Kissing Crush (DK Crush) versus provisional stenting; informs optimal stenting strategy for distal left main bifurcation lesions and clinical outcomes
PRACTICAL-2 (Lavi et al.)	Patients undergoing diagnostic transradial cardiac catheterization	2021	Compares short versus standard durations of radial hemostatic device; informs best practices for hemostasis management and radial artery preservation post-procedure
InnoSEAL-II (Aijaz et al.)	Patients undergoing transradial coronary intervention	2020	Compares combination of InnoSEAL plus TR band versus TR band alone; informs optimal radial hemostasis strategy and radial artery outcomes post-procedure
POST-PCI Functional Testing RCT (Park et al.)	High-risk patients after PCI	2022	Compares routine functional testing versus standard care after PCI; informs the utility and outcomes of post-PCI ischemia assessment strategies
TAILOR-PCI (Pereira et al.)	Patients undergoing PCI	2020	Compares genotype-guided P2Y12 inhibitor selection versus conventional clopidogrel therapy; informs personalized antiplatelet therapy and ischemic outcomes post-PCI
COSIRA Trial (Verheye et al.)	Patients with refractory angina	2015	Evaluates efficacy of a device to narrow the coronary sinus; informs novel therapeutic approaches for patients with refractory angina
BASKET-SMALL 2 (Jeger et al.)	Patients with small coronary artery disease undergoing PCI	2018	Compares drug-coated balloons versus drug-eluting stents; informs non-inferior strategies for treating small-vessel coronary artery disease and reduces stent-related complications
DEBUT Trial (Rissanen et al.)	Patients with de-novo coronary artery lesions and high bleeding risk	2019	Compares drug-coated balloons versus drug-eluting stents; informs safe, non-inferior revascularization strategies in high bleeding risk patients
INFINITY-SWEDEHEART (Erlinge et al.)	Patients undergoing PCI with DynamX bioadaptor vs Resolute Onyx stent	2024	Registry-based randomized trial comparing a bioadaptive stent vs contemporary DES; informs long-term safety and efficacy of next-generation bioadaptor stents
IVUS-ACS (Li et al.)	Patients with acute coronary syndromes undergoing PCI	2024	Compares intravascular ultrasound-guided PCI versus angiography-guided PCI; informs procedural optimization and clinical outcomes for ACS patients

To scale these innovations, practical steps are needed: investment in digital infrastructure that integrates imaging, physiology, and EMR data; cost-effectiveness research to support reimbursement reform; and enhanced education using tools like VR simulation to train operators in advanced techniques. Dedicated IT support and cross-disciplinary collaboration will be critical to sustaining these complex systems. Looking further ahead, the cardiac catheterization laboratory over the next 20–30 years may evolve into a highly data-driven, precision-guided environment. Real-time high-resolution imaging, hybrid physiologic assessments, AI-driven predictive modeling, robotic and automated PCI, and novel bioresorbable technologies could allow procedural decisions to be increasingly tailored to individual patient anatomy, physiology, and genetic risk factors. With thoughtful implementation, precision PCI can evolve from a niche capability into a broadly adopted standard of care that advances the goals of both precision medicine and value-based cardiovascular care.
